# Genetic basis of leg health and its relationship with body weight in purebred turkey lines

**DOI:** 10.3382/ps/pew479

**Published:** 2017-02-23

**Authors:** D. N. R. G. Kapell, P. M. Hocking, P. K. Glover, V. D. Kremer, S. Avendaño

**Affiliations:** * Aviagen Ltd, Newbridge, Midlothian EH28 8SZ, UK; † The Roslin Institute and Royal (Dick) School of Veterinary Studies, University of Edinburgh, Easter Bush, Midlothian EH25 9RG, UK; ‡ Aviagen Turkeys Inc., Lewisburg, West Virginia 24901; § Aviagen Turkeys Ltd, Tattenhall, Cheshire CH3 9GA, UK

**Keywords:** genetic correlation, heritability, leg health, turkey, welfare

## Abstract

The aims of this study were to estimate the genetic parameters for leg and foot health and mobility in purebred turkey lines and their genetic correlations with BW. Traits were gait score (GS) as an overall measure of leg health, footpad dermatitis (FPD), and 2 skeletal leg health traits, namely, valgus and varus deformities (VVD) and tibial dyschondroplasia (TD). Data from 4 different lines, comprising 3 yr of phenotypic records and 4 yr of pedigree information per line, were used. The sex average BW for the lines at 18 wk ranged from 19.1 kg (line A) to 12.4 kg (line D). The prevalence of VVD ranged from 5.2 to 14.6% and for TD from 4.1 to 23.2%. The average score for FPD on a scale of 0 to 100 ranged from 48.5 to 61.1. Gait Score was scored on a scale of 1 to 5, standardized to a mean of 3 and SD of 1. Heritabilities were estimated at 0.08 to 0.13 for GS, 0.01 to 0.07 for VVD, 0.06 to 0.12 for TD, and 0.10 to 0.15 for FPD (all SE ≤ 0.02). Estimates of the genetic correlations between VVD and TD ranged from 0.03 to 0.21 (all SE ≤ 0.08), and estimates of these with GS ranged from 0.07 to 0.87 (all SE ≤ 0.09). The genetic correlations of FPD with GS ranged from 0.00 to 0.34 (all SE ≤ 0.04), and with the skeletal leg health traits from -0.06 to 0.33 (all SE ≤ 0.06). Body weight showed estimated genetic correlations ranging from 0.28 to 0.51 (all SE ≤ 0.06) with GS, -0.06 to 0.50 (all SE ≤ 0.13) with VVD/TD and 0.05 to 0.34 (all SE ≤ 0.05) with FPD. The results suggest that selection for improved leg health can be incorporated effectively in a commercial turkey breeding program using balanced breeding goals, in which production traits and leg health traits are considered simultaneously.

## INTRODUCTION

Leg health is an essential component of modern turkey and chicken breeding programs. Long-term selection for improved leg health has been documented in chickens (Kapell et al., 2012a,b). The breeding programs of Aviagen Turkeys adopted selection for a range of leg health traits, including gait score (**GS**), in the 1970s as an important selection tool in their breeding program. The current breeding program has built on this and developed into an improved, more objective system for scoring traits, with the addition of new traits. Therefore this study focuses on GS as an overall measure of leg health, 2 skeletal leg health traits, namely, valgus and varus deformities (**VVD**) of the long bones and tibial dyschondroplasia (**TD**), as well as the more recently implemented footpad dermatitis (**FPD**) to examine genetic parameters and correlations in 4 contemporary populations.

Gait score is used as an overall assessment of leg health in a range of different species. It generally involves observing the gait of an animal from a specific angle, e.g., from the side or from behind, on an individual basis to evaluate leg structure, posture, and the quality of movement. In poultry it can be used as a tool to assess the welfare at a phenotypic level of a group of birds or an individual (e.g., Dawkins et al., 2009). Whereas some information on gait scoring is available at the phenotypic level, little has been published about its genetic background. At the phenotypic level, Nestor (1984) found a significantly worse walking ability, scored on a subjective 5-point scale, for males in a line selected for BW compared to a random-bred control line. Similarly, Da Costa et al. (2014) found a significant phenotypic correlation between increased BW and a decrease in walking ability in a mixture of genetic lines. Emmerson et al. (1991) found little difference in walking ability among 3 genetic lines selected for different weights, though a better walking ability was found in a control line. They also found a significant gender difference with females having a better walking ability than males. At the genetic level, Quinton et al. (2011) found moderate heritabilities at 0.25 to 0.26 for walking ability. Within the Aviagen Turkeys breeding program, the visual assessment of the gait has been adopted as a selection tool, whereby birds are ranked on their relative performance compared to their contemporaries.

Valgus and varus deformities are 2 common deformities of the long bones, which appear in a range of different species, and are seen as an outwards or inwards angulation, respectively, of the tibiotarsus. They present a major welfare problem because they can lead to walking difficulties and, in severe cases, lameness (Bradshaw et al., 2002). The heritabilities for leg structure (a composite trait including valgus and varus deformities) in turkeys were reportedly low at 0.08 (Quinton et al., 2011). Studies in chickens have found moderate heritabilities for valgus (0.15 to 0.39, Le Bihan-Duval et al. (1997)) and varus (0.21 to 0.30, Le Bihan-Duval et al. (1997)), but low heritabilities for leg angle (0.09 to 0.11, Chen et al. (2011)) and long bone deformities (0.04 to 0.07, Kapell et al. (2012b)).

TD, an abnormal development of the cartilage in the growth plate of the long bones, has been described in turkeys as early as 1967 (McCapes, according to Wise (1975)). A longitudinal study of the prevalence of TD in turkeys found a higher prevalence at 10 and 14 wk of age than at 18 wk (Wilson, 2003). TD lesions may lead to fractures or lameness in meat-type poultry (Bradshaw et al., 2002), although in turkeys no consistent evidence was found that TD was associated with a change in gait (Wilson, 2003). In addition, Hester and Ferket (1998) found no phenotypic link between TD and long bone distortion in male turkeys. In recent decades, technologies have been developed to determine the presence or absence of TD accurately using a low-intensity x-ray imaging scope (Lixiscope). Studies in chickens have shown that TD can be improved effectively through culling of cases as well as selection for superior families based on breeding values (e.g., Wong-Valle et al., 1993; Yalçin et al., 2000; Kapell et al., 2012b), with heritabilities ranging from 0.10 to 0.27 (Kapell et al., 2012b).

Contact dermatitis is an ulceration of the skin, visible as a discoloration that may be accompanied by inflammation and/or necrosis (Greene et al., 1985). Depending on the location, contact dermatitis may appear as, for example, breast blisters or footpad dermatitis. External factors including litter quality (Shepherd and Fairchild, 2010), litter wetness (Martland, 1984; Mayne et al., 2007), and diet (Mayne, 2005) have all been suggested to play a role in the development. Studies have shown that the prevalence at slaughter age in turkeys can be high. Krautwald-Junghans et al. (2011) found that only 4.0% of males and 0.4% of females had clinically normal footpads at 16 wk of age, while Da Costa et al. (2014) found 4.7% normal footpads in males at 16 to 19 wk of age. Studies tended to find a higher prevalence in females (Krautwald-Junghanns et al., 2011; Bergmann et al., 2013). Quinton et al. (2011) estimated very low heritabilities of 0.01 to 0.02 for FPD, whereas studies in broiler chickens have shown a stronger genetic basis, with heritabilities ranging from 0.08 to 0.32 (Kjaer et al., 2006; Ask, 2010; Kapell et al., 2012a).

The aims of this study were to estimate the genetic parameters for a range of different leg health traits and their genetic correlations with BW in 4 purebred turkey lines contributing to commercial crosses from the Aviagen Turkeys breeding program.

## MATERIALS AND METHODS

### Trait Description and Scoring

The data for this study originate from the ongoing recording of leg health traits within the Aviagen Turkeys UK and Aviagen Turkeys US breeding programs. At 14 wk of age (18 wk for line C) males were assessed for TD using a low-intensity x-ray imaging scope (Lixiscope), followed at 17 wk of age by visual assessment of GS/VVD for both sexes (line C males only), and at 18 wk recording of BW and assessment of FPD. All assessments of traits were done by a trained team of scorers, whom are regularly assessed for consistency using correlations between and within scorers. Table 1 gives an overview of the traits included in this study.

**Table 1.** Trait abbreviations, description, and scale of measurement.

Abbreviation	Description	Variable type
BW	Body weight (kg)	Continuous
GS	Gait score	Ordinal, 5 scores
VVD	Valgus or varus type long bone deformity	Binary
TD	Tibial dyschondroplasia: (a) no lesions, (b) moderate lesions, or (c) severe lesions	Ordinal, 3 scores
FPD	Footpad dermatitis: (a) no lesions, (b) mild lesions, (c) moderate lesions, (d) moderately severe lesions, or (e) severe lesions	Ordinal, 5 scores

**Figure 1.**
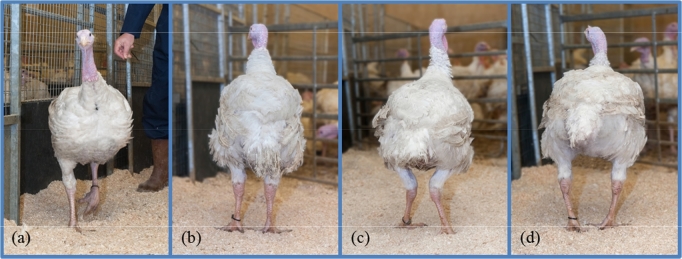
Illustrations of gait scoring, showing (a) the scorer judging the bird as viewed from behind, and (b) healthy legs (score 0) compared to (c) valgus (score 100) and (d) varus deformities (score 100).

GS is a subjective qualitative assessment of a bird in comparison to its contemporaries, scored on a 5-point scale (Table 2). The increasing severity scale ranges from score 1 (above average) to score 5 (below average). The walk of the bird is assessed from behind (as shown in Figure 1a where the scorer stands behind the bird) and scored after considering a range of attributes including the posture, the straightness and angularity of the legs, the stride, whether the bird walks straight without a rocking motion, and the overall fitness of the bird (Figure 1b). A sample of around 30 birds is scored first to determine the baseline performance of the group of contemporaries, after which the whole flock, including the sample, is scored. The aim is to achieve proportions of 5 to 7% for the 2 extreme categories, 44 to 46% for the average category, and 22 to 23% for the remaining 2 categories.

**Table 2.** Description of gait scores.

Score	Description
1	Smooth gait, upright posture, legs straight from hip through hock to foot — superb walk with no defects
2	Smooth gait and upright posture, but perhaps legs not completely straight or slight rocking motion
3	Smooth gait with feet placed straight ahead, but legs may be crooked from hip to hock or hock to foot
4	Halting gait, with perhaps presence of VVD defect and slight to moderate rocking motion
5	Limping gait with side stepping of feet rather than placing feet straight ahead, contributing to poor posture; bird may be unwilling/unable to walk, show the presence of severe VVD defect, or moderate to severe rocking motion

**Figure 2.**
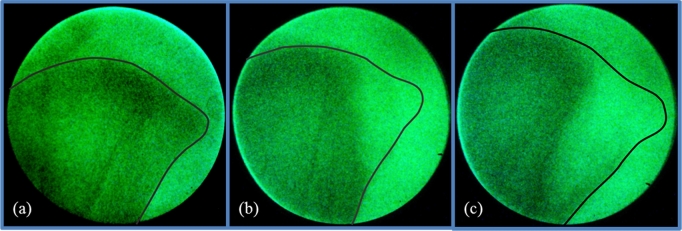
Illustrations of the scores for tibial dyschondroplasia: (a) no lesions (score 0), (b) moderate lesions (score 100), and (c) severe lesions (score 100).

The long bone deformity VVD (valgus - Figure 1c, and varus - Figure 1d) is scored binomially as (0) unaffected or (100) affected — a bird showing either one of the 2 defects is scored as affected. VVD is scored independently of GS, though a bird displaying this defect will never be classified as a score 1 walk. Tibial dyschondroplasia was assessed using the same technique as described in Kapell et al. (2012b) for broiler chickens. Scoring was done using a Lixiscope on a 3-point scale, depending on the extent to which abnormal cartilage developed in the tibia: no lesions, moderate lesions, or severe lesions (Figure 2). The distinction between moderate and severe lesions is recorded for phenotypic monitoring; for the purpose of genetic selection against TD, the focus was on the incidence of affected vs. unaffected birds. For this reason, moderate and severe lesions were combined into one category (score 100) for the genetic analysis, while no lesions were scored as 0. For lines A, B, and C the trait TD is measured only in males that have been identified as selection candidates in a previous step. This step combines breeding values for performance traits and a thorough physical assessment including no clinical prevalence of leg defects prior to 14 weeks. In line D, TD is measured in all males. The 2 defects VVD and TD are considered to be major disorders — any bird showing any of them is culled using a “zero tolerance” policy. This is equivalent to the “zero tolerance” policy used in broiler chickens as described in Kapell et al. (2012b).

**Figure 3.**
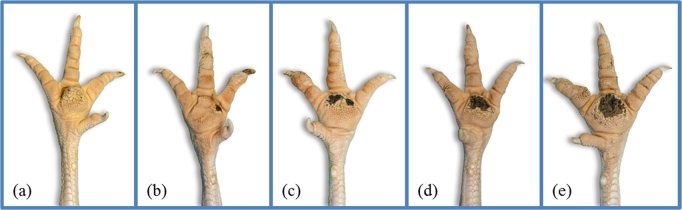
Illustrations of the scores for footpad dermatitis: (a) no lesions (score 0), (b) mild lesions (score 25), (c) moderate lesions (score 50), (d) moderately severe lesions (score 75), and (e) severe lesions (score 100).

FPD was assessed and analyzed on a 5-point scale according to severity of the lesion: no lesions (score 0), mild lesions (up to 25% of the plantar surface affected – score 25), moderate lesions (up to 50% of the plantar surface affected – score 50), moderately severe lesions (up to 75% of the plantar surface affected – score 75), and severe lesions (more than 75% of the plantar surface affected – score 100) (Figure 3). Both feet were evaluated and the higher scoring foot determined the final score.

### Birds, Housing, and Management

All birds were hatched, sexed, and tagged with a barcoded wing band in the hatchery and subsequently moved to the growing farms and distributed over pens according to line. Lines B and D were housed on farms in the United Kingdom and lines A and C in the United States in a highly biosecure pedigree environment where breeding program selection candidates are recorded and selected. Tables 3 and 4 provide a detailed overview of the environmental parameters for the UK and US pedigree environment, respectively, in compliance with UK guidelines and the US National Turkey Federation recommendations. Water and a high-quality diet were provided ad libitum throughout the growing period. Litter was supplied in the form of a layer of wood shavings, which was top dressed as required.

**Table 3.** Description of the environmental parameters in the contemporary population in the United Kingdom. All diets are predominantly wheat-soya based.

Pedigree environment UK			
Parameter/Period	Values		
Feed	Protein g/kg	MJ/kg	Cal./kg
d 0 to 21 - pre-starter	268	11.5	2756
d 22 to 42 - starter	254	11.8	2822
d 43 to 63 - grower 1	229	12.2	2910
d 64 to 91 - grower 2	200	12.5	2987
d 92 to 126 - GP rearer	164	12.8	3060
Stocking density - end of brooding	35.9 to 39.5 kg/m^2^	15.2 to 17.1 birds/m^2^	
Stocking density - end of testing	58 kg/m^2^	2.9 birds/m^2^	
Spot temperature - brooding	32 °C reducing to 19 °C		
Ambient temperature	19 to 23 °C		
Photoperiod	14L:10D		
Light intensity d 0 to 21	min 50 lux		
Light intensity d 22 to final weighing	min 30 lux		

**Table 4.** Description of the environmental parameters in the contemporary population in the United States. All diets are corn-soya based.

Pedigree environment US			
Parameter/Period	Values:		
Feed	Protein g/kg	MJ/kg	Cal./kg
d 0 to 21 - pre-starter crumb	280	12.5	2987
d 22 to 42 - starter crumb	250	12.8	3060
d 43 to 84 - grower 1 pellet	220	13.1	3131
d 85 to 105 - grower 2 pellet	190	13.4	3203
d 106 to final weighing - finisher 1 pellet	170	13.6	3250
Stocking density - end of brooding	34 to 35 kg/m^2^	14 to 17 birds/m^2^	
Stocking density - end of testing	60 kg/m^2^	3 birds/m^2^	
Spot temperature - brooding	31.7 °C reducing to 18.3 °C		
Ambient temperature	20 to 22 °C		
Photoperiod d 0 to 98	14L:10D		
Photoperiod d 99 to final weighing ♂	14L:10D		
Photoperiod d 99 to final weighing ♀	11L:13D		
Light intensity d 0 to 21	min 28 lux		
Light intensity d 22 to final weighing	min 19 lux		

### Statistical Analysis

For the estimation of genetic parameters, the data were restricted to a contemporary population, consisting of circa 3 generations hatched between June 2011 and June 2014 with an extra generation of pedigree (Table 5).

**Table 5.** Statistics for the genetic parameter estimation: number of birds with phenotypic information on at least one trait (Phenotypes), number of birds in the pedigree (Pedigree), number of levels for the permanent environmental effect of the dam (c^2^), and number of levels for the fixed effect accounting for the interaction between the hatch week, pen, and contributing mating group of the individual (Batch).

	Numbers of levels of			
Line	Phenotypes	Pedigree	c^2^	Batch
A	91,499	139,520	6,122	2,422
B	100,087	142,327	7,461	3,259
C	79,011	126,133	2,912	867
D	65,604	103,154	3,085	2,480

All leg health traits were recorded on a binomial or multinomial scale and analyzed using the approach described in Kapell et al. (2012b). For the genetic analysis, the following multiple trait model including 5 traits — BW, GS, VVD, TD, and FPD — was used to estimate genetic parameters:

\begin{equation*}{{\bf y}} = {{\bf Xb}} + {{\bf Za}} + {{\bf Wc}} + {{\bf e}},\end{equation*}

where **y** is the vector of observations of the traits, **b** the vector of the fixed effect accounting for the interaction between the hatch wk, pen, contributing mating group, and the sex of the individual (“batch”), **a** the vector of additive genetic effects, **c** the vector of permanent environmental effects of the dam, and **e** the vector of residuals. **X**, **Z**, and **W** are incidence matrices relating the vectors **b**, **a**, and **c** with **y**. The assumed (co)variance structure was:

\begin{equation*}{\rm{V}}\left[ {\begin{array}{@{}*{1}{c}@{}} {{\bf a}}\\ {{\bf c}}\\ {{\bf e}} \end{array}} \right] = \left[ {\begin{array}{@{}*{3}{c}@{}} {{{\bf A}} \otimes {{\bf G}}}&{{\bf 0}}&{{\bf 0}}\\ {{\bf 0}}&{{{\bf I}} \otimes {{\bf C}}}&{{\bf 0}}\\ {{\bf 0}}&{{\bf 0}}&{{{\bf I}} \otimes {{\bf R}}} \end{array}} \right],\end{equation*}

where **A** and **I** are the additive genetic relationship matrix and identity matrix, respectively. **G**, **C**, and **R** represent the variance and covariance matrices of additive genetic effects, permanent environmental effects of the dam, and residual effects, respectively. Numbers of levels per effect for the bird, permanent environmental effect of the dam, and batch effect are given in Table 5. The variance component analyses were done by restricted maximum likelihood using the software VCE (Groeneveld et al., 2008). The multivariate analysis included the trait BW to remove potential biases in the estimates for leg health traits due to weight associated effects.

## RESULTS

### Descriptive Statistics

Table 6 gives the number of observations, means of BW and GS, and the prevalence of leg disorders, by gender and in total in all lines. It should be noted that, due to a pre-selection step at 14 wk for males in lines A, B, and C, fewer records are available for this sex, and that no GS is recorded in females of line C. The sex-averaged BW ranged from 19.1 kg in line A to 12.4 kg in line D. Males were considerably heavier than females, weighing on average 31 to 32% (lines A and B) or 43% (lines C and D) more than females.

**Table 6.** Descriptive statistics for BW, gait score (GS), valgus and varus deformity (VVD), tibial dyschondroplasia (TD), and footpad dermatitis (FPD) by sex (♂ = male, ♀ = female) and combined (total), for birds hatched between June 2011 and June 2014 (n = number of records, mean and SD in kilograms [BW] or units [GS]; mean score scaled to 0 to 100 for VVD, TD, and FPD).

		Line A		Line B	
Trait	Sex	n	Mean (SD)	n	Mean (SD)
	♂	37,638	22.2 (2.2)	45,846	21.4 (1.7)
BW	♀	52,691	17.0 (1.3)	51,065	16.1 (1.2)
	total	90,329	19.1 (3.1)	96,911	18.6 (3.0)
	♂	38,810	3.1 (1.0)	49,379	3.2 (1.0)
GS	♀	52,689	3.0 (0.9)	50,708	3.0 (0.9)
	total	91,499	3.1 (0.9)	100,087	3.1 (0.9)
	♂	37,638	21.8	45,846	14.7
VVD	♀	52,691	9.5	51,065	8.2
	total	90,329	14.6	96,911	11.3
TD	♂	35,786	16.7	40,850	7.1
	♂	25,471	52.6	33,221	50.1
FPD	♀	36,824	55.4	36,740	55.4
	total	62,295	54.3	69,961	53.1
		**Line C**		**Line D**	
Trait	Sex	n	Mean (SD)	n	Mean (SD)
	♂	29,101	16.3 (1.3)	28,441	15.0 (1.1)
BW	♀	49,910	11.4 (0.9)	37,163	10.5 (0.7)
	total	79,011	13.2 (2.6)	65,604	12.4 (2.4)
	♂	30,276	3.0 (1.0)	28,819	3.1 (1.0)
GS	♀	–		26,905	2.9 (0.9)
	total	30,276	3.0 (1.0)	55,724	3.0 (0.9)
	♂	29,101	13.3	28,441	10.1
VVD	♀	49,910	0.4	37,163	3.6
	total	79,011	5.2	65,604	6.4
TD	♂	6,920	23.2	19,835	4.1
	♂	19,361	62.0	20,296	50.4
FPD	♀	32,011	60.5	26,349	47.0
	total	51,372	61.1	46,645	48.5

The trait GS is scored with the aim of achieving an average of 3 and a standard deviation of 1, which is reflected in the values in this dataset. The prevalence of VVD ranged from 5.2% (line C) to 14.6% (line A). There was a clear contrast between males and females. In line C, due to the low prevalence in females, the contrast between the sexes was greatest; in the other three lines, the males had a 1 to 3 times higher prevalence than females. Due to a pre-selection of the males at 14 wk of age in lines A, B, and C, the prevalence of VVD may be an underestimation compared to the females.

A clear difference in prevalence was found for TD between lines B and D at 7.1 and 4.1% vs. lines A and C at 16.7 and 23.2%, respectively. It should be noted that this is the prevalence in pre-selected males that were free of any other leg defects in all but line D.

**Figure 4.**
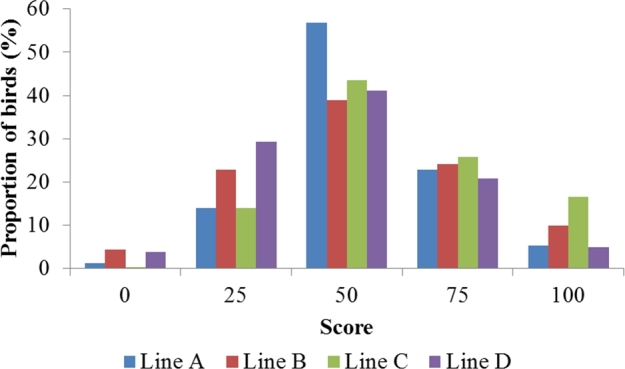
Distribution of the scores for footpad dermatitis.

Figure 4 shows the distribution of FPD prevalence for the 4 lines. The proportion of moderate and low FPD ranged from 57.8 to 74.2% while severe FPD affected between 4.9 and 16.5% of the birds. Females had a tendency to be more affected by FPD, though the percentage of severely affected birds was higher in males.

### Heritabilities

Heritability estimates for BW were moderate in lines A to C (ranging from 0.21 to 0.25) and high in line D (0.43) (Table 7). GS showed a similar heritability for all 4 lines, at 0.08 to 0.13. Estimated heritabilities for VVD were low at 0.01 to 0.07. For TD, estimated heritabilities ranged from 0.06 to 0.12. The trait FPD was generally the most heritable of all leg health traits, at 0.10 to 0.15.

**Table 7.** Heritabilities (bold, diagonal), genetic correlations (above diagonal), and phenotypic correlations (below diagonal) for BW, gait score (GS), valgus and varus deformity (VVD), tibial dyschondroplasia (TD), and footpad dermatitis (FPD). SE as subscript.

	Line A				
Trait	BW	GS	VVD	TD	FPD
BW	**0.25 _0.01_**	0.32 _0.05_	0.18 _0.06_	0.25 _0.02_	0.34 _0.02_
GS	−0.05	**0.10 _0.01_**	0.87 _0.02_	0.07 _0.05_	0.00 _0.04_
VVD	0.00	0.46	**0.07 _0.00_**	0.03 _0.02_	−0.01 _0.04_
TD	0.06	0.01	−0.01	**0.12 _0.01_**	0.05 _0.02_
FPD	0.12	0.00	0.00	0.00	**0.11 _0.01_**
			Line B		
Trait	BW	GS	VVD	TD	FPD
BW	**0.22 _0.01_**	0.51 _0.03_	0.37 _0.05_	0.31 _0.05_	0.05 _0.02_
GS	0.08	**0.12 _0.01_**	0.83 _0.02_	0.27 _0.04_	0.10 _0.02_
VVD	0.03	0.38	**0.04 _0.00_**	0.18 _0.08_	0.04 _0.03_
TD	0.01	0.10	0.03	**0.07 _0.01_**	0.08 _0.02_
FPD	0.09	−0.01	−0.01	−0.05	**0.15 _0.01_**
			Line C		
Trait	BW	GS	VVD	TD	FPD
BW	**0.21 _0.02_**	0.28 _0.06_	0.03 _0.10_	−0.06 _0.13_	0.08 _0.05_
GS	−0.06	**0.08 _0.01_**	0.40 _0.07_	0.48 _0.09_	0.27 _0.04_
VVD	−0.01	0.31	**0.01 _0.00_**	0.16 _0.05_	0.05 _0.05_
TD	0.05	0.07	0.01	**0.11 _0.02_**	0.28 _0.06_
FPD	0.03	0.03	0.01	0.02	**0.10 _0.01_**
			Line D		
Trait	BW	GS	VVD	TD	FPD
BW	**0.43 _0.03_**	0.47 _0.04_	0.50 _0.06_	0.41 _0.09_	0.25 _0.02_
GS	0.07	**0.13 _0.01_**	0.80 _0.03_	0.29 _0.07_	0.34 _0.04_
VVD	0.02	0.30	**0.03 _0.00_**	0.21 _0.06_	0.33 _0.04_
TD	0.01	0.06	0.05	**0.06 _0.01_**	−0.06 _0.05_
FPD	0.12	0.05	0.00	−0.06	**0.15 _0.01_**

### Genetic Correlations Among Leg Health Traits

The estimated genetic correlations of GS with other leg health traits were highest with VVD in most lines (0.80 to 0.87). The only exception was line C, where the genetic correlation was highest with TD at 0.48. The estimated genetic correlations between TD and VVD were low at 0.03 to 0.21. In the 2 heavier lines, FPD showed low genetic correlations with all other leg health traits (between -0.01 and 0.10). In the 2 lighter lines the correlations were higher with all traits (0.27 to 0.34) except VVD in line C (0.05) and TD in line D (−0.06).

At the phenotypic level, all correlations among leg health traits were mostly negligible, except between GS and VVD (at 0.30 to 0.46).

### Genetic Correlations of Leg Health Traits with BW

The genetic correlations of the leg health traits with BW varied considerably between traits and lines. The highest correlations were found for GS with BW (0.28 to 0.51). Line C showed the lowest genetic correlations between BW and the other leg health traits, ranging from -0.06 (TD) to 0.08 (FPD), while the other 3 lines showed moderate genetic correlations for most leg health traits with BW, from 0.18 (VVD in line A) up to 0.50 (VVD in line D). The only exception to this was a low genetic correlation with FPD in line B (at 0.05). At the phenotypic level, all correlations of BW with leg health traits were negligible.

## DISCUSSION

Selection for improved leg health in turkey production systems is of major importance since leg disorders are associated with considerable economic and welfare concerns (e.g., Wise, 1975; Thorp, 1994; Bradshaw et al., 2002). In this paper, we have looked at the genetic parameters for 4 leg health traits and their genetic correlations with BW. Whereas heritabilities are generally low, many genetic correlations are also moderate to low, suggesting that simultaneous improvement of all traits is achievable in broader breeding goals encompassing both health and welfare traits and production traits, in line with other species such as dairy cattle, sheep, and chickens (McKay et al., 2000; Lawrence et al., 2004; Dawkins and Layton, 2012; Kapell et al., 2012a,b).

The genetic parameters in this study were estimated for traits measured at 17 to 18 wk of age (14 or 18 for TD). For males in lines in which an earlier selection step takes places at 14 wk of age, the males are selected based on the same full range of traits as they are selected on at 18 wk. We acknowledge that there is a possible effect of the 14-wk selection on the estimated parameters at 18 wk; however, there are many reasons that individuals do not survive until 18 wk, including mortality and leg defects — in the present study the interest is solely in the observed defects measured until 18 wk as well as the associated production traits at this commercially relevant age.

### Prevalence

Little has been published about the prevalence of VVD in turkeys. The prevalence in this study is lower than the incidence of the composite trait leg structure (Quinton et al., 2011). In their study, 16.7% of the birds in a male line and 16.9% of the birds in a female line showed a form of a leg defect, though the composite trait encompasses a variety of defects including not only valgus and varus deformities.

In the present study TD was measured only on males that were already free from other leg defects in most lines; valgus and varus deformities, as well as lameness, may be the result of underlying TD (Bradshaw et al., 2002); therefore, the prevalence of TD in the birds that were not selected may have been higher.

A direct comparison of FPD in this study with others is not straightforward due to a wide range of different scoring systems that are in use, such as the recommended 5-point classification scheme for FPD in slaughter plants (which formed the basis for the classification system used in this study) (Hocking et al., 2008) or a 7-point classification introduced by Mayne (2007), which has been used in small scale research experiments (e.g., Hocking and Wu, 2013). The prevalence of FPD is in line with previously published results in terms of overall prevalence (Martland, 1984; Krautwald-Junghanns et al., 2011; Allain et al., 2013; Da Costa et al., 2014). In the present study the heavier lines showed a higher average score in females while the lighter lines showed a higher average score in males, consistent with the variation found in other studies — higher in females (Krautwald-Junghanns et al., 2011; Bergmann et al., 2013) vs. higher in males (Clark et al., 2002). However, overall there appears to be a line by sex interaction for FPD, but the exact cause for this is unclear. Environmental factors play a large role in the development of FPD, and measures such as a drier litter material can improve the condition of the feet (Martland, 1984). As a result of their lighter BWT, females were stocked at a lower number of birds/m^2^ than males, which may have affected the litter quality. Also, the difference in diets may have affected the prevalence of FPD, either directly or through its effect on litter moisture.

### Heritabilities

The heritabilities of 0.08 to 0.13 for GS are lower than those found by Quinton et al. (2011) of 0.25 to 0.26, which may be due to the different scoring systems, the fact that the present study, in contrast to Quinton et al. (2011), incorporated a permanent environmental effect of the dam, and the difference in age at which the trait was assessed. Excluding the permanent environmental effect of the dam from the model increased estimated heritabilities to a range of 0.10 to 0.15, suggesting that excluding this effect may lead to an overestimation of the heritability. In addition, GS is a trait that indicates the general fitness of the whole locomotive system of the bird, and there may be a range of different reasons or underlying defects leading to a bird showing a lower than average walking ability. This variability in the factors leading to the observed phenotype may play a role in the low heritability for this trait. Another consideration is the normalization of the trait, which was done to increase accuracy and consistency at recording.

Similar to GS, the heritabilities for VVD obtained in this study are low and in line with heritability estimates for leg structure, hip structure, and foot structure in turkeys of 0.02 to 0.08 (Quinton et al., 2011) and long bone deformities (which include both valgus and varus deformities as well as others) in chickens of 0.04 to 0.07 (Kapell et al., 2012b). This may in part be due to the low prevalence of these defects in all 4 lines; the highest prevalence was seen in line A, which also had the highest heritability at 0.07.

The heritabilities for TD showed a clear difference between lines B and D (0.06 to 0.07) compared with lines A and C (0.11 to 0.12), consistent with differences in incidence. Selection experiments as well as long-term selection in a commercial breeding program in chickens have shown that genetic selection against TD is achievable and can lead to rapid progress in the first years after implementation (Wong-Valle et al., 1993; Kapell et al., 2012b). However, once a significant reduction in prevalence has been achieved, this is likely to be accompanied by a decrease in the heritability, especially since this is a categorical trait analyzed on the observed scale.

All traits included a permanent environmental effect of the dam, which accounted for 2 to 4% of the phenotypic variation in BW, but only up to 2% of the variation in the leg health traits. This is lower than the percentage found in chickens in a range of leg health traits, at 0.5 to 3.6% of the phenotypic variance (Kapell et al., 2012b). This is likely due to the fact that in the present study the age of the birds at the time of measuring the traits was 17 to 18 wk, compared to an age of 5 to 6 wk in chickens. However, while this percentage may seem low, studies comparing models including and excluding maternal effects have shown that omission of the maternal effects may result in an overestimation of the direct heritability (e.g., Koerhuis and Thompson, 1997; Clément et al., 2001; Grosso et al., 2010). To evaluate the effect of the permanent environmental effect of the dam, all 4 models also were run without this effect, which resulted in increased heritabilities for the leg health traits in the range of 0.01 to 0.06.

As pointed out in previous publications by the authors (Kapell et al., 2012a,b) regarding the prevalence and genetic parameters of leg health traits in chickens, the heritability of categorical traits, such as these defect traits, on the observed scale is very much dependent on the number of categories or classifications for a trait, and the frequencies of observations in the different categories. Practical considerations, such as achieving a high repeatability and the need for easily distinguishable categories, mean that genetic parameters and estimated breeding values on the observed scale are favored, but it is acknowledged that the estimated heritabilities in this study are likely to be lower than on the underlying scale or using a threshold model (e.g., Dempster and Lerner, 1950; Gianola, 1982).

### Correlations between Leg Health Traits

With the exception of line C, the estimated genetic correlations between GS and VVD are similar to the genetic correlations for hip structure and leg health with walking ability reported by Quinton et al. (2011) (0.85 to 0.91, compared to 0.80 to 0.87 excluding line C in our study). Line C is also the line with the lowest prevalence of VVD, which may have played a role in the much lower correlation of 0.40. The trait TD showed no clear pattern of genetic correlations with other leg health traits across the lines. In the 3 lightest lines the strongest correlation was with GS, generally followed by the correlation with VVD, which suggests that TD and the walking ability of the bird may have a common genetic basis. However, in the heaviest line there was very little correlation of TD with the other 2 traits, either at the genetic or phenotypic level. Hester and Ferkett (1998) found no difference in incidence of TD between groups of male turkeys with long bone distortions vs. males without, concluding that the long bone distortions exist independently of the presence of TD.

The genetic correlations of FPD with the other 3 leg health traits were low in the 2 heavy lines, but higher in the lighter lines, and all phenotypic correlations were close to zero. In a pairwise correlation between FPD and GS, Da Costa et al. (2014) found only weak to moderate phenotypic correlations between them, and the correlation disappeared in a multiple linear regression that included also BW, litter score and season.

### Correlations between Leg Health and BW

Nestor (1984) reported an unfavorable genetic correlation of BW with lateral deviations of legs and with walking ability, although this was apparent only in the last 4 generations of a 16-generation selection period. At the phenotypic level, Emmerson et al. (1991) found no significant difference in walking ability among 3 genetic strains selected for increased BW, leg weight, or shank diameter, but all 3 strains showed a poorer average walking ability than a control line. More recently, Quinton et al. (2011) reported unfavorable genetic correlations with BW, ranging from 0.26 to 0.41 for hip structure and 0.31 to 0.49 for leg structure, though phenotypic correlations were very low (0.05 to 0.08 for hip structure and 0.04 to 0.13 for leg structure). Da Costa et al. (2014) noted that at the phenotypic level, BW had a weaker influence on GS and FPD than the environmental factor of litter quality. The present study found weaker unfavorable genetic correlations with VVD than with GS in all but the lightest line. Birds affected by a leg disorder may be less vigorous, leading to a negative environmental correlation between BW and leg health traits, which in turn would explain a much higher genetic than phenotypic correlation (Mercer and Hill, 1984). In the present study the environmental correlation between BW and GS or VVD was consistently negative, down to -0.12.

At the phenotypic level FPD generally showed the strongest correlation with BW of all leg health traits of up to 0.12, but at the genetic level the correlation was of only a moderate magnitude up to 0.34. Lines A and D, with the higher genetic correlations with BW, are the 2 lines with a lower proportion of severely affected birds compared to lines B and C. This moderately unfavorable correlation in 2 lines in this study is in line with estimates from Quinton et al. (2011) at 0.23 to 0.24.

In conclusion, selection for improved leg health can be incorporated in a commercial turkey breeding program. While heritabilities were generally low, genetic correlations with a production trait were also low to moderately unfavorable. The evidence from the current research suggests that a stringent culling policy of selection candidates showing any sign of leg disorders, combined with predicted breeding values that allow the identification of those families that are prone to develop leg disorders, will contribute to the continuous improvement of leg health. Broad and balanced breeding goals, considering all production traits and leg health traits simultaneously, are essential to ensure that progress is achieved in all traits, despite somewhat antagonistic correlations among them.
